# Detection of Salt Content in Canned Tuna by Impedance Spectroscopy: A Feasibility Study for Distinguishing Salt Levels

**DOI:** 10.3390/foods13111765

**Published:** 2024-06-04

**Authors:** Inés Zabala, Santos Merino, Unai Eletxigerra, Jorge Ramiro, Miren Burguera, Estibaliz Aranzabe

**Affiliations:** 1 Tekniker, Basque Research Alliance (BRTA), 20600 Eibar, Spain; ineszabala26@gmail.com (I.Z.); jorge.ramiro@tekniker.es (J.R.); miren.burguera@tekniker.es (M.B.); estibaliz.aranzabe@tekniker.es (E.A.); 2 Departamento de Electricidad y Electrónica, Universidad del País Vasco, UPV/EHU, 48940 Leioa, Spain

**Keywords:** impedance spectroscopy, canned tuna, saline solutions, equivalent electric circuit

## Abstract

The electrical impedance of dilute aqueous solutions containing extracts from five brands of canned tuna is analyzed using impedance spectroscopy in order to analyze their salt content and detect the potential presence of other salts beyond the well-stated NaCl. A complex electrical impedance is modeled with an equivalent electrical circuit, demonstrating good agreement with experimental data. This circuit accounts for the contribution of ions in the bulk solution, as well as those contributing to electrode polarization. The parameters describing the equivalent circuits, obtained through fitting data to the electrical impedance, are discussed in terms of the various ion contributions to both the electrical double layer at the electrode interface and the electrical conductivity of each solution. The ionic contribution to the electrical impedance is compared with that of a pure NaCl solution at the same concentration range. This comparison, when extended to real samples, allows for the development of a model to estimate the electrical conductivity of canned tuna samples, thereby determining the salt concentration in tuna. The model enables differentiation among the various samples of tuna studied. Subsequently, the potential presence of other ions besides Na^+^ and Cl^−^ and their contribution to the electrical properties of each canned tuna extract is considered, especially for samples with a higher ratio of the sum of K^+^ and phosphates to Na^+^ concentration. This analysis shows the potential of impedance spectroscopy for on-site and rapid analysis of salt content and/or detection of additives in canned tuna fish.

## 1. Introduction

The dispersion of permittivity in water finds an explanation through the Debye equations, which offer a relatively simple framework. These equations were originally developed for dielectrics where molecules neither interact nor form intermolecular hydrogen bonds, a condition not entirely applicable to liquid water. Nevertheless, despite this limitation, Debye’s theory aligns well with experimental data and serves as a valuable tool for interpreting dielectric studies, particularly of polar solvents like water. Thus, in the exploration of electrical properties across complex fluids such as water [1], liquid crystals [2], ionic solutions [3], and ionic liquids [4], researchers have delved into understanding the interactions between solute and solvent molecules. In electrolyte solutions, these interactions encompass the interplay between ions themselves, ions and solvent, as well as undissociated electrolyte molecules, solvent, and ions, all primarily governed by electrostatic forces. The strength of these interactions hinges on the dielectric properties of the surrounding medium and the spatial arrangement of ions and molecules. These interactions induce intricate kinetics in various aspects, including hydrogen bonding, the interface between water and substrates, and the formation of ion pairs, which in turn shield water dipoles and orient them around ions. This phenomenon initially decreases the dielectric permittivity of the sample. However, it competes with other factors, such as the disruption of hydrogen bonds and the reduction in intermolecular space in aqueous solutions containing ions [5]. This competition increases dipole fluctuation and, consequently, the dielectric permittivity of the sample. This complex behavior finds elucidation when observed over time scales that correspond to these processes. For instance, the high-frequency range in the gigahertz region offers insights into the dynamic reorientation of water molecules, whereas lower frequency ranges below 1 MHz provide information on ion dynamics and electrical conductivity.

Electrical Impedance Spectroscopy (EIS) is a technique used to study the dynamic contributions of molecules in different mediums including ions dissolved in water. EIS consists of applying an external low amplitude sinusoidal voltage, of variable frequency, between two electrodes containing the ionic solution and measuring the current flowing in the circuit. Thus, the complex electrical impedance is given by the generalized Ohm law:

(1) $$Z\;(\omega )=V_{0}\exp(i\omega t)/i(t)$$

where V_0_ is the amplitude of applied voltage, ω = 2πf, is the angular frequency, and i(t) is the electrical current. Therefore, the complex electrical impedance will depend on the frequency, f, the sample concentration, and the dynamic behavior of the sample, including molecule rotations, the electrical charge of solved ions, electrical conductance, and the temperature of the medium. The measured impedance spectra, usually fitted with an equivalent electrical model, represent an electrical fingerprint of the sample, providing an insight into its properties and behavior. Thus, EIS up to 1 MHz has been largely used to analyze the electrical properties of ion-based water solutions as a function of ion species, analyzing the relationship between electrical impedance and ion concentrations [6,7]. However, this analysis is typically hindered by a significant limitation common to all conductive solutions. When subjected to an electric field, ions have a propensity to migrate toward the electrode/sample interface, giving rise to the formation of an ionic double layer (DL) in these regions. In such layers, the applied voltage dissipates rapidly, resulting in extensive electrical polarization of the material and minimal electric field penetration into the bulk sample at low frequencies [8]. Consequently, the capacitance of these layers can overshadow the signal from the bulk sample relaxation at lower frequencies. This phenomenon, known as electrode polarization, profoundly impacts the interpretation of dielectric spectra [9].

EIS has been used for assessing food quality and has shown a high potential for real-time use because changes in the complex impedances of food provide information about its chemical composition and help to follow changes in its state [10]. Thus, EIS has been extensively used to characterize milk [11] and yogurts [12]; to detect the quality and adulteration of dairy products such as bovine milk [13]; to differentiate between oliva varieties [14], to monitor the ripening of fruit [15]; to discriminate between fresh and frozen-thawed fish [16]; and to identify changes in pork during refrigerated storage [17]. EIS has been also extensively applied for agricultural product quality detection [18].

The aim of this study is to utilize impedance spectroscopy to investigate the electrical conductivity of canned tuna samples, establishing correlations with the predominant salt concentration, sodium chloride (NaCl), added to the tuna, while also considering the potential presence of other significant ion concentrations. Previously, impedance spectroscopy has been employed as a rapid and portable technique to differentiate between tuna samples that were frozen, refrigerated, or supercooled [19] as well as to forecast nutritional degradation in tuna samples over time [20]. However, there are no references to its utilization in predicting salt content.

Tuna fish, as long-lived organisms, tend to accumulate pollutants such as essential metals (Zn or Mn), as well as highly toxic metals like Hg, Pb, and Cd, in addition to essential minerals such as K, Ca, and P [21]. Conversely, commercial practices involve controlling, adding, and preserving moisture throughout the process of capturing, processing, distributing, storing, and preparing canned tuna. Consequently, the use of polyphosphates and phosphate salts, such as tetrasodium diphosphate, tetrapotassium diphosphate, potassium tripolyphosphate, dipotassium phosphate, potassium metaphosphate, or pentasodium triphosphate, has been reported [22,23,24]. These salts bind tuna muscle proteins, enhancing water retention and preserving muscle integrity, thereby preventing color changes due to oxidation. Moreover, NaCl is often added to canned tuna to lower water activity, inhibiting microbial growth and extending shelf life. In this context, impedance spectroscopy can offer precise insights into controlling the NaCl content in both raw and cooked tuna before canning. Additionally, it may provide indications regarding the potential addition of other salts aimed at either long-term preservation or color retention.

In this study, various brands of canned tuna with differing salt contents were analyzed by impedance spectroscopy, employing standard conductive measurements and inductively coupled plasma (ICP) as technical analytical references. The study correlates electrical impedance behavior across frequencies with an impedance equivalent circuit associated with a physical model describing the behavior of ions in solution [3]. We employed a step-by-step approach using samples of increasing complexity. This methodology enabled us to validate specific electrochemical models and isolate the contributions of different components in the analysis of real samples. Initially, the model was validated using pure NaCl water solutions and an equivalent electrical circuit previously described to study the electrical impedance behavior of KCl and NaCl water solutions [3]. This allowed us to validate the model using disposable screen-printed carbon electrodes and to compare the peculiarities of this setup with a traditional three-electrode laboratory setup. It also allowed us to determine the cell constant associated with the cell geometry and electrode configuration, which was then used for subsequent analysis of canned tuna samples. We also studied doped NaCl samples to ensure the model could account for the presence of additional ions beyond those from NaCl alone. Finally, the model was validated with processed canned tuna samples, providing a method to measure NaCl concentration in tuna through frequency-dependent electrical impedance analysis.

## 2. Materials and Methods

### 2.1. Sample Preparation

Five different brands of canned tuna were analyzed. The canned tuna contains natural tuna which has not been mixed with oils and only water and salts are identified as ingredients by each brand. The extract from each canned tuna was analyzed using the next protocol specifically developed for this study. After opening the can, the whole amount of tuna was taken and dried in a glass overnight at 100 °C. Afterward, ultrapure deionized water with a resistivity of 18.2 MΩ cm at 25 °C was added to the tuna sample up to a volume of 50 mL, and the sample was shaken by means of a magnetic stirrer for 10 min. The volume was then passed through a paper filter adding ultrapure water to complete a 100 mL sample.

For validating the model with additional interferents, these samples were mixed with NaCl (Sigma-Aldrich, (Saint Louis, MO, USA) purity > 99%) to prepare different salt concentrations. For this protocol, 1 mL samples were mixed with 0.5 mL of NaCl with different concentrations between 0.05 and 1.01% (*w*/*w*) to get different salt concentration samples. Samples were shaken and stored at 4 °C for impedance spectroscopy measurement.

### 2.2. Impedance Spectroscopy

The custom-made Screen-Printed Carbon Electrodes (SPCEs), consisting of a circular carbon working electrode (4 mm diameter, WE), a carbon counter electrode (CE), and a silver pseudo reference electrode (RE), were acquired from Metrohm Dropsens (Oviedo, Spain). The specific cable connector (DRP-CAS), used as an interface between the SPCEs and the impedance analyzer, was also acquired from Metrohm Dropsens.

A UV/ozone modification of the SPCEs was carried out to improve its electron transfer rate and electrical performance [25]. A ProCleaner™ Plus UV/Ozone cleaner (BioForce Nanosciences, Ames, IA, USA) in which a 10 mW/cm^2^ Hg bulb lamp provided 185 and 254 nm UV light was used. The lamp was warmed up for 5 min, and then SPCE strips were placed in the chamber and modified at a 10 cm distance from the Hg lamp for 5 min. In the process, oxygen molecules absorbed 185 nm UV light and formed ozone, then ozone molecules absorbed 254 nm UV light and broke into atomic oxygen.

**Figure 1:**
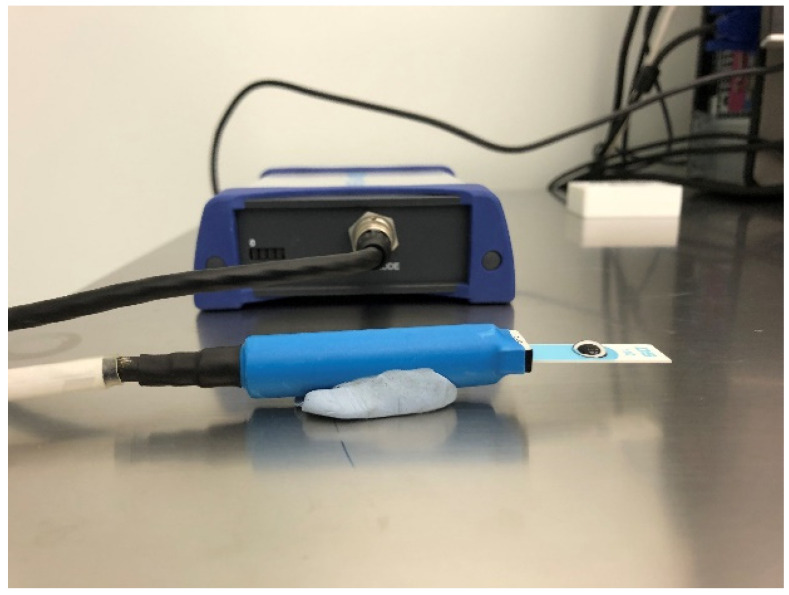
Setup used for measurements. showing detail of electrode surface and sample drop deposited onto its surface for impedance spectroscopy measurements.

All the electrode measurements were performed using a µStat-I 400s portable impedance analyzer from Metrohm Dropsens after the UV/ozone modification of the SPCEs. The analyzer is designed to work on a broad spectrum range between 1 mHz and 1 MHz, with an AC amplitude range between 1 mV and 0.35 V (RMS). It has a resolution of 1 mV in the applied potential, with an intensity current accuracy of ≤0.5% over the whole current range between 1 nA and 100 mA. The impedance spectroscopy measurement was performed directly by pipetting an 80 µL sample drop on the electrode’s surface (Figure 1). The impedance spectroscopy signal was obtained by applying a sinusoidal potential with an amplitude of 10 mV and a frequency range between 100 mHz and 100 kHz, taking five points for each decade. The measurements were performed at room temperature (21–23 °C) after the sample had been preserved at this temperature for 12 h. For each sample, the impedance measurement was performed three times with an interval of 10 min between each measurement. With this procedure, the reproducibility was guaranteed with a maximum deviation of 0.5%.

The measured EIS spectra were fitted to an equivalent circuit using the DropView 8400 software tool from Metrohm Dropsens. This software exploits a nonlinear least square regression to fit data with the function obtained from the equivalent circuit, using the damped least-square method (DLS). The fitting procedure changes the values of the parameters until the mathematical function matches the experimental data within a certain error margin. The goodness of the fit is represented by the χ^2^ (chi-squared) value which is minimized for the best fitting selecting reasonable starting values.

### 2.3. Inductively Coupled Plasma Detection

Compositional analysis was determined by ICP-optical emission spectrometry (ICP-OES) in a Horiba Ultima Expert spectrophotometer. The sample solution was pumped into the back end of a glass concentric nebulizer by a peristaltic pump, and a radiofrequency-induced argon plasma with a 40.68 MHz frequency was used to interact with the aerosol. A thermally stabilized optical system, comprising a plane grating monochromator with a focal length of 1 m, was used, while a groove density of 2400 groves per millimeter and High Dynamic Detectors (HDDTM) were used for atomic line identification.

### 2.4. UV-VIS Spectrophotometry

Chloride concentration was analyzed using a DR6000 UV-VIS spectrophotometer from Hach equipped with a Hach Lange kit LCK 311 (Hach Lange, Düsseldorf, Germany). For analysis, samples of 0.1 mL in volume were pipetted into the test cuvettes, shaken, and inserted into the cuvette slot placed in the spectrophotometer device for analysis at a 468 nm wavelength.

### 2.5. Conductivity Measurement

The electrical conductivity of the samples, prepared as specified in Section 2.1, was measured using a laboratory conductivity meter (Crison EC-Meter GLP 31 (Crison Instruments, Barcelona, Spain)) with automatic temperature compensation at 25 °C.

### 2.6. Statistical Analysis

The data collected in this study were analyzed using appropriate statistical methods to ensure the validity of the test. A total of three measurements for each sample, with an interval of 10 min between them, were performed to assess the impedance values. KaleidaGraph 4.0 and Matlab statistical software R2018a were used for data analysis and graphical representation. Linear curve fitting and linear correlation coefficients were performed using KaleidaGraph. Chi-square values, representing the sum of the squared errors between the experimental data and the fitted equivalent circuit, were calculated using the DropView 8400 software from Metrohm Dropsens.

## 3. Results and Discussion

### 3.1. Sample Analysis by Standard Techniques

Live tuna fish contain around 0.25% of their weight in NaCl, and this percentage increases when they are stored in freezers in contact with seawater before being stored on land. Afterward, the tuna is cooked and mixed with NaCl or a combination of NaCl and potassium phosphates to enhance its flavor, avoid the spoilage of the fish, and keep an attractive color for consumers. The level of added salts may depend on the NaCl concentration found in the raw tuna fish, which is largely the most abundant salt found in natural canned tuna.

Five different brands of canned tuna films were studied, and ICP-OES and UV-VIS spectroscopy were used to analyze the different ions and their respective concentrations in the samples while electrical conductance was measured for each brand. Table 1 shows the contribution of the most relevant ions found. Ions with concentrations below 1 mM, such as Ca^2+^ and Mg^2+^, were omitted.

**Figure 2:**
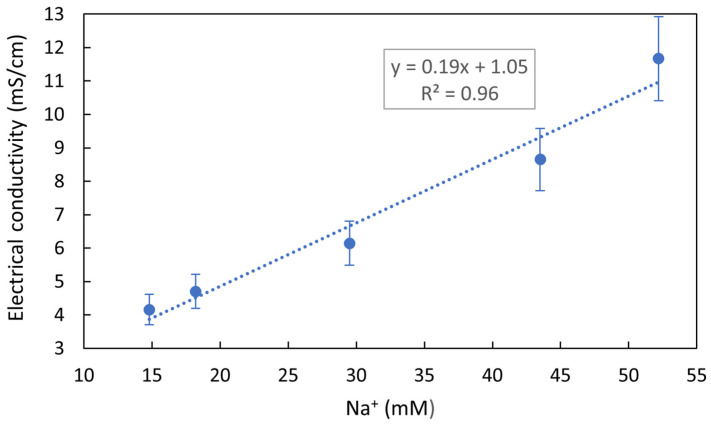
Experimental data and linear curve fitting between the sodium ion concentration measured by ICP-OES for each commercial tuna sample and its electrical conductivity.

The extracted tuna sample compositions, analyzed by ICP-OES and UV-VIS spectrophotometry, should show an almost equimolar concentration for Na^+^ and Cl^−^. This is because NaCl penetration has been reported in meat [26] and fish [27] processing, as studied by Energy-Dispersive X-ray Spectroscopy (EDS) and Magnetic Nuclear Resonance, respectively. These studies show that during salting, both ions diffuse at the same speed. Small variations may be attributed either to the fact that the concentration of Cl^−^ ions in seawater is slightly higher than Na^+^ concentration [28], or to fish tissue composition, which could affect Na^+^ and Cl^−^ retention [22]. However, this late statement is not applicable to processed tuna in which there are no differences between different muscles or organs. Cl^−^ ion concentrations were not well detected by ICP-OES, as halogens are not easy to excite in a plasma source, and because their emission wavelengths providing yield good sensitivity are low (at 133–134 nm) and challenging for conventional optics and solid-state detectors [29]. Thus, the Cl^−^ ion concentration was analyzed by UV-VIS spectroscopy while Na^+^ concentration was considered as a reference for the comparison of NaCl solutions due to the superior accuracy of the ICP-OES tool and the higher Cl^−^ concentration at sea. The concentration of Na^+^ obtained from the different brand samples analyzed by ICP-OES was studied versus the electrical conductivity measured by an electrical conductivity meter, and a linear relationship was obtained (Figure 2).

### 3.2. Reference Samples: Analysis of NaCl-Based Solutions by Impedance Spectroscopy

From the range of concentrations of interest, different solutions of NaCl in ultrapure water were prepared in the 2–85 mM range and analyzed by impedance spectroscopy to be used as control and comparison with real samples. Nyquist plots at low concentrations revealed two main regions: a semicircle related to the liquid bulk contribution and a tail-like segment related to the electrode DL phenomena. As the NaCl concentration increases, the bulk liquid semicircle is not observed due to the increased ionic conductivity of the solution.

Considering the lack of a simple theoretical model to analyze the EIS experimental data in ionic solutions, equivalent electrical circuits are used to reproduce experimental data from ionic dilute aqueous solutions. A simple equivalent circuit describing the physical behavior consists of a Debye circuit, composed of a capacitor associated with bulk capacitance, in parallel with a resistor related to the ionic conductivity in the bulk. It is connected to a constant phase element (CPE), which describes the DL of ions associated with the electrode polarization [30]. This last CPE term is a capacitive element with a frequency-independent negative phase between current and voltage which interpolates between a capacitor and a resistor. There is also a similar element with a positive phase angle, which is called inductive CPE. Both the capacitive and inductive CPEs are members of the family of fractional circuit elements, or fractance [31,32]. They have been largely applied to characterize the DL impedance in the study of highly conductive suspensions [33] in which an equivalence impedance following a power-law is proposed:

(2) $$Z_{CPE}\left(\omega \right)=\frac{A}{{\left(i\omega \right)}^{n}}$$

where *A* is a function of the applied voltage, the geometrical parameters of the electrodes, the self-diffusion coefficient of the ions in the solutions, and the bulk ions concentration; and *n* is a parameter ranging between 0 and 1 for the capacitive CPE and between −1 and 0 for inductive CPE. This impedance term, originally introduced to explain the finite slope of the Nyquist plot, has been largely studied in terms of the associated physical models [8]. Among them, a well-accepted model describes the electrical polarization as an over-damped oscillator [32]. In this model, the ions are oscillating near the interface, and the motion is over-damped with a dissipating force that is a function of different mechanisms such as ion collisions or the friction force describing the interaction between ions and the solution. In this model, the ions behave as a spring-mass system, thus performing damped and forced harmonic motion. The over-damping oscillator model proposed the introduction of a complex viscosity approach [34] with some equivalence with an anomalous diffusion model close to the surface electrode, showing very similar behavior to that predicted by the capacitive CPE impedance. In this model, A is a reciprocal amplitude depending on mass, charge, number of ions, and the damping behavior of the oscillator, and n is a parameter varying between 0 and 1 showing an ideal capacitive behavior or resistive behavior as n = 1 or 0, respectively. This model describes the so-called fractal dimension [8] in which n changes with the kind of ions in solution, the temperature, and its concentration, and in which a minimal change in n depends on both the surface characteristics of the electrode and the type of solution that is in contact with the electrode. Thus, for the same substrate, the fitting parameters depend on the properties of the ions in the solution, and it confirms the relevance of the mixture of ions in the DL formation.

**Figure 3:**
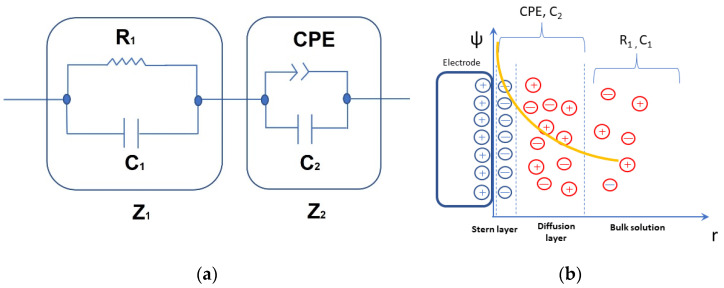
Diagram of equivalent electric circuit proposed to fit the electrical impedance of different solutions (**a**) and scheme showing the relationship between the impedance components of the equivalent circuit describing the system and its variation as a function of the distance to the electrode surface (**b**). The superimposed curve shows the screening of the electric potential ψ due to the electrode polarization.

Considering this approach, the equivalent circuit represented in Figure 3a was used to fit both the Bode and Nyquist diagram, in which Z_1_ represents the impedance of a Debye circuit where C_1_ is associated with the bulk capacity and R_1_ is inversely proportional to the bulk electrical conductance:

(3) $$Z_{1}\left(\omega \right)=\frac{R_{1}}{1+i\omega R_{1}C_{1}}$$

The Z_2_ component represents the interface impedance due to electrode polarization. This approach leads to a local coupling of the ohmic resistance to the interfacial capacitance, which has been stated as a necessary condition for CPE-like behavior in interfacial phenomena [35]. It is composed [3] of one capacitor C_2_ connected in parallel with a CPE as described in Figure 3a, giving an electrical impedance:

(4) $$Z_{2}\left(\omega \right)=\frac{A{\left(i\omega \right)}^{-n}}{1+{\left(i\omega \right)}^{1-n}{AC}_{2}}$$

It shows ideal capacitor behavior when n = 1, denoting the electric DL capacitance, while it behaves as a parallel circuit composed of electrical resistance A and a capacitor C_2_ when n = 0. These last elements are associated with the mobile diffuse layer and the capacitive contribution C_2_ is associated with the Stern layer, representing the immediate ion layer on the electrode [36,37]. It describes how ions on the electrode surface are governed by an electrostatic attraction which is balanced by the thermal energy in a diffusive transport of ions (Figure 3b).

**Figure 4:**
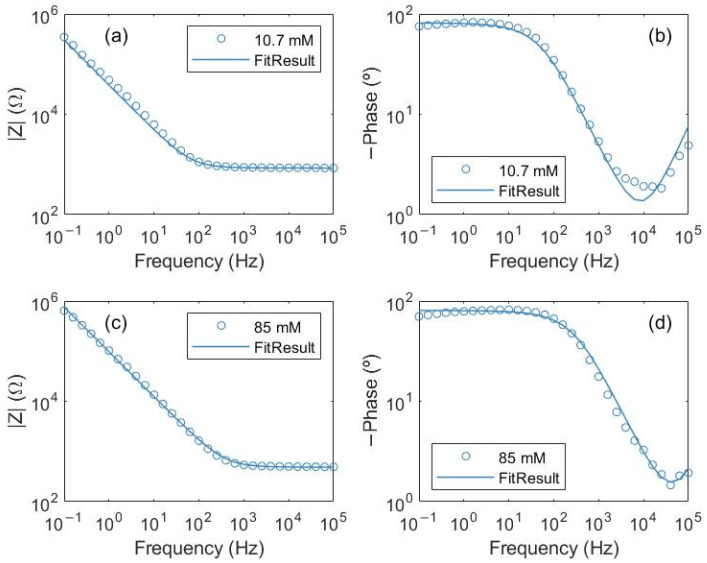
Bode diagrams for NaCl solutions and equivalent electric circuit proposed fitting. NaCl solutions with concentrations: (**a**,**b**) 10.7 mM and (**c**,**d**) 85 mM.

**Figure 5:**
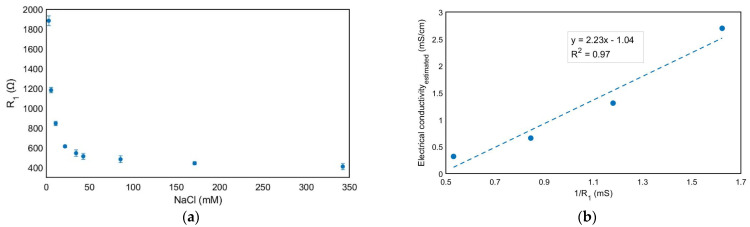
R_1_ values for different NaCl concentrations obtained from the fitting to the equivalent circuit of the EIS measurements. The mean value ± standard deviation is shown for n = 3 independent experiments (**a**) and estimated electrical conductivity and proposed linear curve fitting according to Kohlrausch’s law for low range concentrations (2.6–22 mM) of NaCl solutions with respect to the inverse of R_1_ obtained from fitting the equivalent circuit to the EIS measurements (**b**).

Fitting the experimental impedance spectroscopy to Z(ω) = Z_1_(ω) + Z_2_(ω) (Figure 4), the R_1_ values for the different NaCl solutions conductivities were determined (Figure 5a) in which two higher concentrations of salt were added to check the saturation of free ions. Table 2 shows how the R_1_ and n parameters change with the different concentrations while the parameter A is in the range of 10^6^ Ωs^−n^ and C_1_ and C_2_ are in the range of 10^−10^ F for the NaCl studied solutions.

The electrical conductance of the low concentration range of solutions (up to 22 mM) was determined by Kohlrausch’s law [38], which describes the electrical behavior of low-concentration electrolytes in solutions, stating that the limiting molar conductivity of an electrolyte can be represented as the sum of individual contributions of the anions and cations of the electrolytes. Considering this approach, the electrical conductivity can be expressed as:

(5) $$\sigma =\sum_{i}{ᴧ}_{m,i}^{0}C_{i}$$

where ${ᴧ}_{m,i}^{0}$ is the limiting molar conductivity of ion I and $C_{i}$ is its concentration. Thus, Figure 5b shows the estimated electrical conductivity in this low-concentration regime and it is represented versus the inverse of R_1_ figures, obtained from the impedance spectroscopy measurements. A linear relationship was obtained, and the slope of this line represents the electrode cell constant [39] k = 2.2 cm^−1^ (Figure 5b). This value agrees with the geometry of the electrodes, from which a cell constant of 1 cm^−1^ is estimated considering a simple approach consisting of considering a 1 mm distance between the working electrode area (0.125 cm^2^) and the counter electrode (0.1 cm^2^). The experimental data also provide good agreement as compared with the referenced electrical conductivity (1 mS/cm) for a standard solution of 8.4 mM [40], showing 2.4 mS/cm as the R_1_ value, and a cell constant for a 10 mM solution is considered.

### 3.3. Analysis of Real Samples Doped with NaCl Using Impedance Spectroscopy

**Figure 6:**
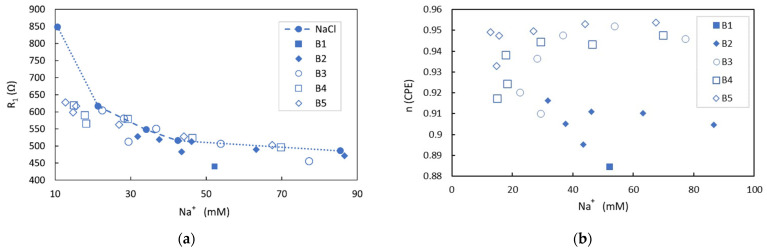
Ionic electric resistances in solution (R_1_) of extract tuna samples in NaCl solutions obtained from the fitting to the equivalent circuit of the EIS measurements in which as a guide to the eye, NaCl referenced solutions are included as a dotted line (**a**). B1 sample was not mixed with NaCl solution, and data included corresponds to the pure extract sample. Behavior of n parameter for different mixtures of extract tuna samples and NaCl solutions as obtained from the fitting to the equivalent circuit of the EIS measurements (**b**).

Electrical impedance measurements were developed for the five different brands of tuna extract in different concentrations of NaCl, while Na^+^ concentration was analyzed by ICP-OES. The fitting of the equivalent circuits provides the values of the ionic electrical resistances in solution (R_1_), which are represented along with those found for the reference NaCl solutions in water (Figure 6a). It can be observed that for similar concentrations of Na^+^, and considering the experimental error, the electrical resistance of solutions is smaller than that predicted for the same concentration of NaCl in water. This fact is more clearly noted at low Na^+^ concentrations, while these differences tend to be almost negligible as the concentration of Na^+^ is increased in the matrix. This denotes the presence of additional ions contributing to the electrical conductivity. Particularly, this behavior is more clearly observed for B1, B4, and B5 samples in which the ratio of the sum of K^+^ and phosphates ions concentrations to Na^+^ concentration is higher (B1: 0.81, B2: 0.72, B3: 0.43; B4: 0.93; B5: 0.89) while it is hardly appreciated in B3 samples with a lower proportion of the cited added ions.

Additionally, the n parameter obtained from the fitting to the equivalent circuit is represented in Figure 6b, in which the tendency for a fixed brand is a slight increase with the Na^+^ concentration until a certain plateau is reached. For a given concentration, the n parameter changes slightly with the different extract samples. The n parameter depends on the electrode surface and the ions that are in contact with the electrode, as well as its size, concentration, and the ion hydration shell. In the over-damped oscillator model used to describe the electrode polarization, the dissipating force is a function of the collision interactions, which is also related to these same variables. In a more physical model to explain the electrode polarization, it can be described as the formation of a layer of adsorbed ions along the electrode surface due to the migration of free ions in solution under the influence of an electric field. This layer will attach to a further layer of an oppositely charged ion arranging the electrical DL. This arrangement of layers drives the potential drop over the diffuse layer, and the characteristic length, referred to as the Debye length, of this exponential decay (Figure 3b) is given by the Debye–Huckel expression [41]. This states that it is proportional to $\sqrt{T/\sum_{i}n_{i}}$, where T is the temperature and $\sum_{i}n_{i}$ is the sum of the ions’ concentration. According to the ion concentration detected by ICP-OES and following this simple model, the characteristic length, in the range of a few nanometers, should be lower for samples containing higher ionic strength, increasing the electrical DL capacitance [37]. For the analyzed tuna samples, the n parameter is close to one, denoting an almost capacitive behavior of the CPE. For values of n of one, the electric DL behaves as an ideal capacitor, and an estimation of the equivalent capacitor can be extracted from Z_2_(w). Considering that the A and C_2_ values obtained from the spectrum fitting are around 10^6^ Ω s^−n^ and 10^−10^ F, respectively, the electric DL capacitance is in the range of 8 µF/cm^2^. This value is in good agreement with the most common range of electric DL capacitances reported in the literature, which are in the range of 5–20 µF/cm^2^ [42] or 6–12 µF/cm^2^ [37], as NaCl concentration changes over five orders of magnitude between 10^−5^ and 1 M.

### 3.4. Real Extract Tuna Analysis by Impedance Spectroscopy

**Figure 7:**
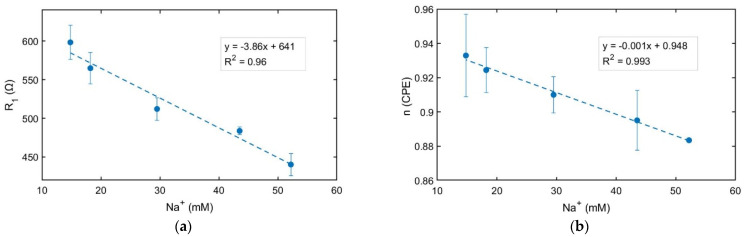
Electrical resistivity of the different extract samples B1–B5 obtained from the EIS measurements (**a**); Variation of n parameter from the fitting to the equivalent circuit for the same samples (**b**). A linear curve fitting is shown for (**a**,**b**). The Na concentration is given by the ICP-OES analysis of each canned tuna sample. The mean value ± standard deviation is shown for each concentration for n = 3 independent experiments.

The different extract samples were analyzed separately, analyzing their electrical resistivity as a function of the Na^+^ concentration obtained from the ICP-OES data. A linear relationship was obtained between both parameters, as the average of three replicates was studied for each concentration and brand (Figure 7a). It denotes that Na^+^ concentration correlates well with the solution’s electrical resistivity, and EIS measurements may provide an easy way to determine the Na^+^ concentration in a canned tuna sample. The electrical resistivity of the various tuna extracts exhibited statistical significance in distinguishing between any pair of tuna samples (the *t*-test sample showed a *p*-value varying from 0.001 to 0.02 for a 95% confidence level). Additionally, the n parameter was also evaluated for each brand, showing a linear relationship between this parameter and the Na^+^ concentration (Figure 7b). Although no significant differences were detected for the n parameter, Figure 7b showed a lower n parameter for higher Na^+^ concentrations of the extract tuna samples. As the DL is characterized by changes in electrical charge density in comparison with its value in the volume of the sample, it may be initially hypothesized that the contribution of sodium ions in each sample plays a relevant role in the composition of the DL interface between the sample and electrodes.

On the other hand, the electrical conductivity for each canned tuna sample can be estimated from the electrical resistivity obtained from the EIS measurements and the cell constant: B1: 5.0 mS/cm; B2: 4.55; mS/cm; B3: 4.29 mS/cm; B4: 3.89 mS/cm; and B5: 3.67 mS/cm. These figures fall within the same range as the direct conductivity measurements included in Table 1, exhibiting the same decreasing trend observed in measurements obtained through the standard technique.

### 3.5. From Macroscopic Data to a Microscopic Model of Ions in Water-Based Solutions

A microscopic model of water can be employed to interpret these results, focusing on the behavior of ions in solution. The significant difference in electronegativities between hydrogen and oxygen atoms in water leads to the formation of a dipole and electrostatic interaction between adjacent water molecules. This interaction results in the creation of hydrogen bonds, which are weak bonds with low dissociation energy. Therefore, at room temperature, hydrogen bonds can break and reform continuously on a timescale ranging from femtoseconds to picoseconds [43]. When studying an ionic solution at high potentials, electrostatic forces dominate, and the accumulation of ions at the interface is governed by the electrostatic interaction between the ions and the surface, disregarding specific ion-water and water-water interactions. Consequently, the hydration shells of ions are depleted at the electrode interface [44]. However, when small voltages are applied, as in this case, the ions’ hygroscopic behavior significantly influences the composition of the DL at the surface, and the structure of the hydration shell becomes highly specific to individual ions [45]. Here, the behavior of ions is determined by factors such as their size, charge density, and associated atomic entropy and free energy of hydration. Thermodynamic studies were conducted to assess the ease with which ions can be hydrated, and free energies were tabulated, allowing for the description of changes in the reorientation times of water molecules during hydration [46]. This led to the conclusion that Na^+^ and K^+^ ions cause significant disruption to the water structure, favoring the orientation of hydrogen-bonded molecules around them, while Cl^−^ ions can be accommodated without causing dramatic distortions to the water network [47,48]. According to these thermodynamic estimations, Na^+^ is expected to be more strongly anchored to hydration due to its higher charge density compared to Cl^−^ or K^+^, and the free energy required to remove one hydration water molecule from the hydration layer of Na^+^ is higher than that for Cl^−^ or K^+^. The timescale for this process is typically in the range of picoseconds [44]. Therefore, within the timescale studied by impedance spectroscopy, hydrogen bonds are continuously broken and ions are dynamically intercalated, resulting in a significant alteration of the water network. At the level of DL composition and disregarding the varying feasibility of ion adsorption onto the electrode, which is macroscopically governed by the C_2_ component, all these processes are expected to influence the behavior of the CPE due to the different ion concentrations present in the solution. Specifically, this will affect both the resistive and capacitive behavior of the CPE, resulting in a change in the n parameter. This effect is clearly observed in Figure 7b, where an increase in Na^+^ concentration across different samples correlates with a reduction in the n parameter, indicating a more resistive behavior of the diffusion layer in the DL. These data do not conclusively determine which ion (Na^+^, Cl^−^, or K^+^) has a stronger influence on this parameter. However, it is hypothesized that Cl^−^ may have a greater impact, as it can intercalate into the water network without significantly distorting it within the Debye length [45,46].

In summary, the impedance spectroscopy-based model proposed can predict the bulk conductivity of the solution, indicating the qualitative presence of additional ions besides NaCl. This model offers a solution for determining the NaCl concentration in tuna through impedance spectroscopy sample analysis. Moreover, the capability of various ions to alter the molecular network of water and their interaction with water molecules at the electrode interface level may afford impedance spectroscopy the ability to predict the qualitative presence of specific ions.

The resolution of impedance spectroscopy is not yet on par with well-established techniques such as ICP-mass spectroscopy, typically utilized in large laboratory facilities for detecting toxic ions derived from Cu, Pb, or Hg in canned tuna. However, the protocol employed by impedance spectroscopy for detection is compatible with portable and rapid on-site readouts. Impedance spectroscopy operates via a label-free transduction mechanism, avoiding the need for reagents to enhance the signal. While certain reagents can enhance sensitivity and specificity for detection, they often lead to a more complex and costly detection process. For instance, gold nanoparticles can be used to detect color changes via UV-vis spectroscopy, associated with alterations in surface plasmon resonance properties as they aggregate in the presence of specific fungicides in canned tuna [49]. Similarly, metal-organic frameworks with high affinity to Hg can be employed to detect toxic Hg^2+^ ions in canned tuna fish [50].

Additional research in impedance spectroscopy could benefit from the implementation of chemometric models, particularly as real samples doped with potassium and phosphate ions are studied alongside their potential to alter the water molecular structure at the electrode interface. This advancement could lead to a more quantitative technique. Furthermore, extending the spectroscopic range up to gigahertz frequencies could aid in examining changes in the dynamics of water molecules in the bulk as they hydrate different ions, thereby supporting the establishment of calibration curves.

## 4. Conclusions

Impedance spectroscopy was utilized to analyze various brands of canned tuna samples, presenting a rapid and feasible protocol for measuring the NaCl concentration. The experimental results were interpreted through an equivalent electrical circuit model consisting of two sets of circuits connected in series. One set represents the bulk behavior of charges in solution, while the other is associated with charge accumulation on the electrode surface. The fitting parameters extracted from the model, along with their relationship with the physical model of electrode polarization, enable the estimation of the electrical conductivity of solutions and may suggest the presence of additional ions beyond those provided by the NaCl salt. These ions may reflect the composition of the tuna, but they could also indicate the potential addition of other salts during storage, processing, and cooking before canning. Impedance spectroscopy offers an alternative to conventional lab techniques and can be employed prior to canning tuna to regulate the salinity level, thereby affecting its taste, preservation, and properties.

Further research aimed at advancing quantitative detection by impedance spectroscopy should capitalize on its label-free detection method, exploiting the absence of specific molecules or reagents. Enhancing specificity for the targeted ions and molecules is crucial. This involves conducting multivariate calibration using a diverse range of samples doped with varying concentrations of various salts, and these models should be reinforced by data obtained from standard laboratory techniques, similar to those employed in this research. The presence of multiple ions in solutions poses significant interference, necessitating a rigorous calibration process. These findings could be extrapolated to other food matrices processed under varied protocols and conditions, including storage, processing, or cooking. The EIS holds promise for advancing towards pocket-sized sensors for on-site food quality monitoring, provided certain obstacles can be addressed. One such challenge is the variation in impedance signal with temperature, which may require offset correction for large dynamic temperature ranges. On the other hand, simplifying sample preparation as much as possible is crucial for obtaining detailed information quickly. Therefore, it is essential that the electrodes are designed to accommodate both solid and liquid types of food, taking their shapes into account. They should also ensure proper sample penetration, particularly for solid-based samples.

## Figures and Tables

**Table 1:** Electrical conductance and ionic concentration measured by ICP-OES and UV-Spectrophotometry for five brands of canned tuna. The mean values of three independent experiments are provided for each ion, with standard deviations falling approximately within 1% of the mean for Na^+^, K^+^, and phosphate ions, and within 5% for Cl^−^ ions. The mean values and standard deviations of the conductivity for each sample are provided based on three independent measurements.

Ionic Concentration (mM)	B1	B2	B3	B4	B5
Cl^−^	48.5	50.2	37.2	30.4	20.8
Na^+^	52.2	43.5	29.5	18.2	14.8
K^+^	22.2	17.9	6.6	8.8	6.5
Phosphates	21.0	13.6	6.1	8.0	6.7
**Conductivity (mS/cm)**	11.67 ± 1.26	8.65 ± 0.93	6.14 ± 0.66	4.7 ± 0.51	4.16 ± 0.45

**Table 2:** R_1_ and n parameters obtained from fitting experimental data to the described equivalent circuit with NaCl-based solutions. The mean value +/− standard deviation is shown for n = 3 independent experiments.

NaCl (mM)	R1 (Ω)	n (CPE)
2.6	1884 ± 49	0.95 ± 0.01
5.3	1183 ± 27	0.95 ± 0.00
10.7	847 ± 22	0.90 ± 0.01
22.0	616 ± 14	0.95 ± 0.02
34.2	547 ± 32	0.94 ± 0.04
42.5	515 ± 30	0.89 ± 0.01
85.0	485 ± 35	0.89 ± 0.01
171.1	445 ± 17	0.94 ± 0.02
342.2	411 ± 30	0.93 ± 0.03

## Data Availability

The original contributions presented in the study are included in the article, further inquiries can be directed to the corresponding author.

## References

[1] Lenzi E.K., Fernandes P.R.G., Petrucci T., Mukai H., Ribeiro H.V. (2011). Anomalous diffusion approach applied to the electrical response of water. Phys. Rev. E.

[2] Mrukiewicz M., Perkowski P., Mazur R., Chojnowska O., Piecek W., Dabrowski R. (2016). Strong modulation of electric permittivity at an isotropic-nematic phase transition in a liquid crystal mixture for optical devices based on the Kerr effect. J. Mol. Liq..

[3] Lima L.F., Vieira A.L., Mukai H., Andrade C.M.G., Fernandes P.R.G. (2017). Electrical Impedance of aqueous KCl and NaCl solutions: Salt concentration dependence on components of the equivalent circuit. J. Mol. Liq..

[4] Cassol T.M., Duarte A.R., Ribas C.S., Fernandes P.R.G. (2021). Dielectric characterization of new task ionic liquid crystals with carboxyl groups by means of impedance spectroscopy from 10 mHz to 10 MHz. J. Mol. Liq..

[5] Buchner R., Hefter G. (2009). Interactions and dynamics in electrolyte solutions by dielectric spectroscopy. Phys. Chem. Chem. Phys..

[6] Duarte A.R., Batalioto F., Barbero G., Figueiredo Neto A.M. (2014). Measurement of the impedance of aqueous solutions ok KCl: An analysis using an extension of the Poisson-Nernst-Planck model. Appl. Phys. Lett..

[7] Grossi M., Parolin C., Vitali B., Ricco B. (2019). Electrical impedance spectroscopy (EIS) characterization of saline solutions with a low-cost portable measurement system. Eng. Sci. Technol. Int. J..

[8] Ishai P.B., Talary M.S., Caduff A., Levy E., Feldman Y. (2013). Electrode polarization in dielectric measurements: A review. Meas. Sci. Technol..

[9] Aoki Koichi J. (2016). Frequency-dependence of electric double layer capacitance without Faradaic reactions. J. Electroanal. Chem..

[10] Guermazi M., Fendri A., Kanoun O., Derbel N. (2018). Potential of impedance spectroscopy for real-time assessing of food quality. IEEE Instrum. Meas. Mag..

[11] Lopes A.M., Machado J.A.T., Ramalho E., Silva V. (2018). Milk Characterization Using Electrical Impedance Spectroscopy and Fractional Models. Food Anal. Methods.

[12] Freitas A.C., Morais L.C., Oliveira M.M., Pinto S.M., Vilela J. (2023). Application of electrical impedance spectroscopy for the characterisation of yoghurts. Int. Dairy J..

[13] Alana Minetto T., Denardi B., Da Silva G., Amarante A., Cazonatto A., Da Silva W. (2022). Identifying adulteration of raw bovine milk with urea through electrochemical impedance spectroscopy coupled with chemometric techniques. Food Chem..

[14] Luna J.M.M., Luna A.M., Fernández R.E.H. (2020). Characterization and Differentiation between Olive Varieties through Electrical Impedance Spectroscopy, Neural Networks and IoT. Sensors.

[15] Grossi M., Ricco B. (2017). Electrical Impedance Spectroscopy (EIS) for biological analysis and food contamination: A review. J. Sens. Sens. Syst..

[16] Fuentes López A., Masot Peris R., Fernández Segovia I., Ruiz Rico M., Alcañiz Fillol M., Barat Baviera J.M. (2013). Differentiation between fresh and frozen-thawed sea bream (Sparus aurata) using impedance spectroscopy techniques. Innov. Food Sci. Emerg. Technol..

[17] Leng Y., Sun Y., Wang X., Hou J., Zhao X., Zhang Y. (2020). Electrical impedance estimation for pork tissues during chilled storage. Meat Sci..

[18] Cheng J., Pengpeng Y., Huang Y., Zhang G., Lu C., Jiang X. (2022). Application status and prospect of impedance spectroscopy in agricultural product quality detection. Agriculture.

[19] Kang T., Shafel T., Lee D., Lee C.J., Lee S.H., Jun S. (2020). Quality retention of fresh tuna stored using supercooling technology. Foods.

[20] Teixeira L.G., Bertemes F.P., Cox M.K. (2021). Fish quality investigations using electrical impedance spectroscopy: Preliminary results. J. Phys. Conf. Ser..

[21] Ormaza-González F.I., Ponce-villao G.E., Pin-Hidalgo G.M. (2020). Low mercury, cadmium and lead concentrations in tuna products from the easter Pacific. Heliyon.

[22] De Almeida Lemos L.L., Goncalves A.A. (2019). Can pH and water-to-protein ratio be good instruments to evaluate the abusive water added in seafood by phosphate addition?. J. Aquat. Food Prod. Technol..

[23] Lampila L.E. (1993). Functions and Uses of Phosphates in the Seafood Industry. J. Aquat. Food Prod. Technol..

[24] Goncalves A.A. (2012). Phosphates for Seafood Processing. Phosphates: Sources, Properties and Applications.

[25] Wang J., Xu Z., Zhang M., Liu J., Zou H., Wang L. (2019). Improved of electrochemical performance of screen-printed carbon electrodes by UV/ozone modification. Talanta.

[26] Filgueras R., Peyrin F., Venien A., Henot J.M., Astruc T. (2016). Sodium chloride difussion during muscle salting evidenced by energy-dispersive x-ray spectroscopy imaging. J. Agric. Food Chem..

[27] Geonzon L.C., Yuson H.A., Takahashi K., Matsukawa S. (2021). Study on salinity pemetration process into fish meat by simulation and MRI. Food Sci. Technol..

[28] Millero F.J., Feistel R., Wright D.G., Mc Dougall T.J. (2008). The composition of Standard Seawater and the definition of the Reference-Composition Salinity Scale. Deep. Sea Res. Part I Oceanogr. Res. Pap..

[29] HORIBA Scientific Application Note—Analysis of Chlorine, Bromine and Iodine in Water Using ICP-AES. https://static.horiba.com/fileadmin/Horiba/Application/Water/Drinking_Water_Utilities/Analysis_of_Chlorine__Bromine_and_Iodine_in_water_using_ICP-OES.pdf.

[30] Sanabria H., Miller J.H. (2006). Relaxation processes due to the electrode-electrolyte interface in ionic solutions. Phys. Rev. E.

[31] Bonatti R.S., Meyer Y.A., Padilha G.S., Bortolozo A.D., Osório W.R. (2020). Silicon content affecting corrosion behavior of Al_P_/Si_P_ composites in a biodiesel blend. Corrosion.

[32] Holm S., Holm T., Martinsen O.G. (2021). Simple circuits equivalents for the constant phase element. PLoS ONE.

[33] Bordi F., Cametti C., Gili T. (2001). Reduction of the contribution of electrode polarization effects in the radiowave dielectric measurements of highly conductive biological cell suspensions. Bioelectrochemistry.

[34] Silva Arthur E.T., Andrade Thiago M., Freire Fernando C.M. (2017). Overdamped oscillator model with a complex viscosity to interpret impedance spectroscopy data. J. Phys. Chem. C.

[35] Hirschorn B., Orazem M.E., Tribollet B., Vivier V., Frateur I., Musiani M. (2010). Determination of effective capacitance and film thickness from constant-phase-element parameters. Electrochim. Acta.

[36] Macdonald J.R. (1987). Impedance spectroscopy and its use in analyzing the steady-state AC response of solid and liquid electrolytes. J. Electroanal. Chem. Interfacial Electrochem..

[37] Khademi M., Barz D.P.J. (2020). Structure of the electrical double layer revisited: Electrode capacitance in aqueous solutions. Langmuir.

[38] Harned H.S., Owen B.B. (1964). The Physical Chemistry of Electrolytic Solutions.

[39] De la Rica R., Sánchez C.F., Baldi A. (2006). Polysilicon interdigitated electrodes as impedimetric sensors. Electrochem. Commun..

[40] Hach Certified Conductivity Standard Solution, 1015 µS/cm, 0.05% NaCl, 500 mL|Hach United Kingdom—Overview|Hach. https://uk.hach.com/1015-s-cm-certified-reference-material-crm-conductivity-standard-solution-0-05-nacl-500-ml/product?id=24929257897.

[41] Debye P., Huckel E. (1923). The theory of electrolytes: I. Lowering of freezing point and related phenomena. Phys. Z..

[42] Lu M., Beguin F., Frackowiak E. (2013). Supercapacitors: Materials, Systems and Applications.

[43] Martiniano H.F.M.C., Galamba N. (2013). Insights on hydrogen-bond lifetimes in liquid and supercooled water. J. Phys. Chem. B.

[44] Alfarano S.R., Pezzotti S., Stein C.J., Lin Z., Sebastiani F., Funke S., Hoberg C., Kolling I., Ma C.Y., Mauelshagen K. (2021). Stripping away ion hydration shells in electrical double-layer formation: Water networks matter. Proc. Natl. Acad. Sci. USA.

[45] Mancinelli R., Botti A., Bruni F., Ricci M.A., Soper A.K. (2007). Hydration of sodium, potassium, and chloride ions in solution and the concept of structure maker/breaker. J. Phys. Chem. B.

[46] Marcus Y. (1994). Viscosity B-coefficients structural entropies and heat capacities, and the effect of ions on the structure of water. J. Solut. Chem..

[47] Roy S., Pal B. (2019). Comparison of structure making/breaking properties of alkali metal ions Na^+^, K^+^ and Cs^+^ in water. arXiv.

[48] Marcus Y. (2009). Effect of ions on the structure of water structure making and breaking. Chem. Rev..

[49] Heleyel M., Elhami S. (2019). Sensitive, simple and rapid colorimetric detection of malachite green in water, salmon and canned tuna samples based on gold nanoparticles. J. Sci. Food Agric..

[50] Singh S., Numan A., Zhan Y., Hung T.V., Nam N.D. (2020). A novel highly efficient and ultrasensitive electrochemical detection of toxic mercury (II) ions in canned tuna fish and tap water based on a copper metal-organic-framework. J. Hazard. Mater..

